# Serum Proteins, HMMR, NXPH4, PITX1 and THBS4; A Panel of Biomarkers for Early Diagnosis of Hepatocellular Carcinoma

**DOI:** 10.3390/jcm11082128

**Published:** 2022-04-11

**Authors:** Jung Woo Eun, Jeong Won Jang, Hee Doo Yang, Jooyoung Kim, Sang Yean Kim, Min Jeong Na, Eunbi Shin, Jin Woong Ha, Soyoung Jeon, Young Min Ahn, Won Sang Park, Suk Woo Nam

**Affiliations:** 1 Department of Pathology, College of Medicine, The Catholic University of Korea, Seoul 06591, Korea; jetaimebin@gmail.com (J.W.E.); jykim0201@nate.com (J.K.); sangyean90@catholic.ac.kr (S.Y.K.); mjna@catholic.ac.kr (M.J.N.); eunb0513@catholic.ac.kr (E.S.); hajo1997@naver.com (J.W.H.); jeonsy1997@catholic.ac.kr (S.J.); wonsang@catholic.ac.kr (W.S.P.); 2 Department of Gastroenterology, Ajou University School of Medicine, Suwon 16499, Korea; 3 Department of Internal Medicine, The Catholic University of Korea College of Medicine, Seoul 06591, Korea; garden@catholic.ac.kr; 4 Liver Cirrhosis Clinical Research Center, Seoul 06591, Korea; 5 Functional RNomics Research Center, The Catholic University of Korea, Seoul 06591, Korea; yhd9020@catholic.ac.kr; 6 NEORNAT, Inc., Seoul 06591, Korea; 7 Department of Biomedicine & Health Sciences, Graduate School, The Catholic University of Korea, Seoul 06591, Korea; 8 Department of Kidney System, College of Oriental Medicine, Kyung Hee University, Seoul 02447, Korea; omdan@hanmail.net

**Keywords:** liver cancer prediction, serum biomarker panel, multistage hepatocarcinogenesis, signal peptide, early diagnosis

## Abstract

The high morbidity rate of hepatocellular carcinoma (HCC) is mainly linked to late diagnosis. Early diagnosis of this leading cause of mortality is therefore extremely important. We designed a gene selection strategy to identify potential secretory proteins by predicting signal peptide cleavage sites in amino acid sequences derived from transcriptome data of human multistage HCC comprising chronic hepatitis, liver cirrhosis and early and overt HCCs. The gene selection process was validated by the detection of molecules in the serum of HCC patients. From the computational approaches, 10 gene elements were suggested as potent candidate secretory markers for detecting HCC patients. ELISA testing of serum showed that hyaluronan mediated motility receptor (HMMR), neurexophilin 4 (NXPH4), paired like homeodomain 1 (PITX1) and thrombospondin 4 (THBS4) are early-stage HCC diagnostic markers with superior predictive capability in a large cohort of HCC patients. In the assessment of differential diagnostic accuracy, receiver operating characteristic curve analyses showed that HMMR and THBS4 were superior to α-fetoprotein (AFP) in diagnosing HCC, as evidenced by the high area under the curve, sensitivity, specificity, accuracy and other values. In addition, comparative analysis of all four markers and AFP combinations demonstrated that HMMR-PITX1-AFP and HMMR-NXPH4-PITX1 trios were the optimal combinations for reaching 100% accuracy in HCC diagnosis. Serum proteins HMMR, NXPH4, PITX1 and THBS4 can complement measurement of AFP in diagnosing HCC and improve identification of patients with AFP-negative HCC as well as discriminate HCC from non-malignant chronic liver disease.

## 1. Introduction

Hepatocellular carcinoma (HCC) is the fifth most common type of cancer diagnosed and the second leading cause of death worldwide. Primary liver cancer includes HCC (comprising 75~85% of cases) and intrahepatic cholangiocarcinoma (comprising 10~15% of cases) as well as other rare types [1,2]. The incidence of HCC and mortality rates of HCC patients are constantly increasing, according to Global Cancer Statistics 2020 [1]. For all countries, the 5-year survival rate is less than 5% [3,4]. This dismal outcome is partly due to the lack of accurate biomarkers for timely diagnosis. As a consequence, only 30~40% of HCC patients are diagnosed in time for potentially curative treatments [5].

Most HCCs develop in patients with chronic liver diseases, particularly those with chronic hepatitis (CH) or liver cirrhosis (LC) and, as with other cancers, are characterized by an obviously multistep process in tumor progression [6]. Chronic hepatocyte destruction is triggered by a number of genetic alterations, and reprogramming can bring on abnormal growth and progression in small nodular hypercellular lesions named dysplastic nodules (DNs) [7]. These pre-malignant lesions progress into early-stage HCC (eHCC) that is characterized as a small, differentiated HCC of uncertainly nodular type and then into advanced HCC that is defined as a clearly nodular character and frequently microvascular invasion [8]. According to current knowledge of multistep hepatocarcinogenesis, patients at high risk of HCC development are closely monitored, and a lot of small uncertain regions are identified by diagnostic analyzers. However, eHCC reveals minimal atypia and lacks definite metastatic or aggressive proliferation. Because of the above reasons, findings of a diagnostic marker that will improve histological diagnosis of eHCC and suitable treatment are eagerly expected [9].

Currently, AFP and abdominal ultrasound of liver are the most broadly utilized tools for HCC diagnosis. Liver ultrasonography is certainly an efficient approach for HCC detection with a sensitivity of 60~90% and specificity of above 90% [10]. However, eHCC exhibits a lack of invasive or abnormal growth, and although improvement in imaging technologies can make detect small nodular lesions less than 1 cm, performing repeated liver ultrasonography and other imaging modalities is economically burdensome [11]. The other diagnostic arm is serum AFP at a level of 20 ng/mL, which is usually used as the upper limit of normal. However, this biomarker suffers from low sensitivity (25~65%), particularly for the detection of early-stage HCC [12,13]. In addition to low sensitivity, the poor specificity inherent in only using AFP as a diagnostic screening tool for HCC is reflected in the many other medical conditions that can lead to elevated serum levels, including 15~58% of patients with CH and 11~47% with LC, responsible for high false positive rates [12,13,14]. Therefore, novel and reliable diagnostic biomarkers to complement AFP are needed to make clinical outcomes better.

Global characterization of the molecular aberrations in both chronic disease and pre-cancerous regions of HCC should accelerate the development of biomarkers for early diagnosis and risk stratification, as well as the recognition of preventive interference to change or block the progression of cancer. Despite the expanded number of studies regarding profiling advanced stage tumors, studies concerning profiling of the genetic alteration rarely focus on chronic liver disease or pre-cancerous tissues. To overcome this deficit, we performed whole transcriptome RNA sequencing using next-generation sequencing (NGS RNA-seq) of 108 human hepatic tissues comprising fresh normal liver, CH, LC, DNs and multistep HCCs that were analyzed by hepatopathologists (Catholic_mLIHC, GSE114564). We then established a candidate gene selection strategy to find potential secretory peptides or proteins by filtering genes with signal peptide cleavage sites from within the HCC-specific molecular signature. After gene selection, the gene was validated using ELISA analysis of molecules in liver patients’ serum. From this, four molecules (HMMR, NXPH4, PITX1 and THBS4) were suggested as an HCC biomarker panel with superior capability (compared to AFP alone) in detecting of liver tumor potential for HCC patients. Here, we suggest that collectively, four novel serum markers, HMMR, NXPH4, PITX1 and THBS4, exhibit good diagnostic performance for the early diagnosis of HCC.

## 2. Materials and Methods

### 2.1. Study Design and Patient Cohort

**Figure 1:**
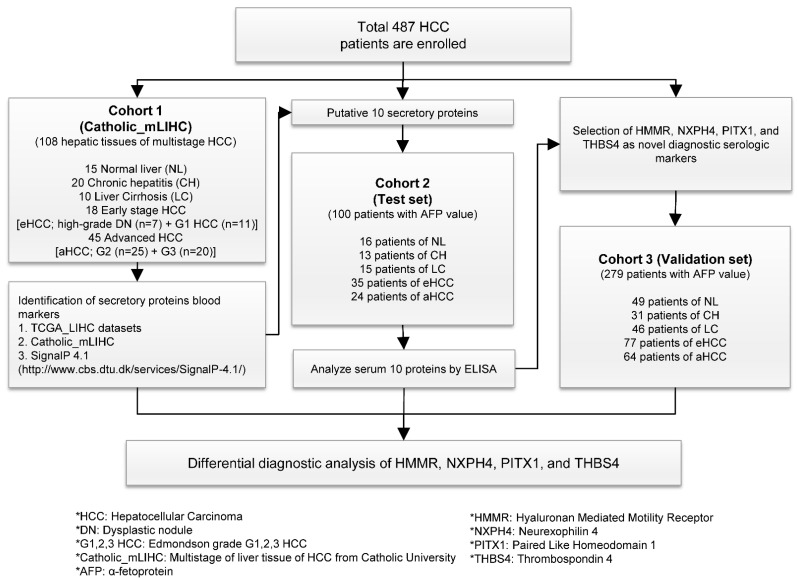
Strategy to identify novel serum markers for hepatocellular carcinoma.

Three independent cohorts of patients with liver disease were involved in this study. Cohort 1 (108 snap-frozen tissues from 86 HCC patients) was used for whole transcriptome NGS RNA-seq analysis. Cohort 2 (771 samples from 100 patients) was used as a test set for performing ELISAs (enzyme-linked immunosorbent assay) on the 10 candidate serum markers, and cohort 3 (1148 samples from 279 patients) was used as a validation set for the four potential serum markers (Figure 1). Written informed consent was obtained from all subjects in accordance with the Declaration of Helsinki, and the study was approved by the Institutional Review of Board (IRB) of the Songeui Campus of the Catholic University of Korea College of Medicine (IRB approval No: MC12EISI0106, MC12SNMI0184).

### 2.2. NGS RNA-Seq Data Analysis

For the large-scale NGS RNA-seq analysis, total RNA was extracted from frozen liver tissues of cohort 1 patients using the TRIzol reagent (Invitrogen, Waltham, MA, USA). RNA quality control was performed with the Agilent Bioanalyzer system (Agilent Technologies, Santa Clara, CA, USA). The sequencing library was prepared with the Truseq Stranded Total RNA Sample Preparation Kit (Illumina, San Diego, CA, USA), followed by a library quality check using the Agilent Bioanalyzer system. Sequencing was performed on Illumina HiSeq2000 machines (Illumina) using the standard Illumina RNAseq protocol with a read length of 2 × 100 bases. All sequenced reads were quality checked using FastQC followed by mapping to the human reference genome (hg19) and the Ensembl version 73 gene annotation using STAR software version 2.6. To compare expression between genes within samples, gene expression was estimated using Cuffquant and Cuffnorm packages from Cufflinks. Gene abundances were normalized by library and gene length by calculating fragments per kilobase of exon per million mapped reads (FPKM). The raw data have been uploaded in the Gene Expression Omnibus (GEO) database (Accession Number: GSE114564) of the National Center for Biotechnology Information (NCBI).

### 2.3. Publicly Available Genomic Data Analysis

To recapitulate the expression level of selected marker gene elements in HCC patients, genomic data were obtained from The Cancer Genome Atlas liver hepatocellular carcinoma project (TCGA_LIHC) and the GEO database of the NCBI (Accession Number: GSE6764). Level 3 mRNA expression data of TCGA_LIHC RNA-seq V2 were log^2^ transformed [log^2^(RSEM+1)] and used to assess the gene expression levels.

### 2.4. Prediction of Secretory Proteins

The program SignalP 4.1 (http://www.cbs.dtu.dk/services/SignalP-4.1/ (accessed on 25 April 2016)) was used for selecting gene elements harboring signal peptide cleavage sites. The SignalP 4.1 software run online with parameters indicated in the organism group: eukaryotes; D-cutoff values: default; method: input sequences do not include TM regions. According to the manual, SignalP 4.1 is the same package as SignalP 4.0 except that some formatting options have been added.

### 2.5. Assessment of Serologic Marker Proteins by ELISA

Serum obtained from patients was centrifuged at 3000× *g* for 15 min and stored at −70 °C until testing. All the serum samples were thawed and assayed for 10 putative serologic markers, cyclin B2 (CCNB2), DNA replication factor Cdt1 (CDT1), Cochlin (COCH), CUB and Sushi multiple domains 1 (CSMD1), HMMR, NXPH4, Olfactomedin-like protein 29 (OLFML2B), PITX1, THBS4 and ubiquitin conjugating enzyme E2 T (UBE2T) using commercially available ELISA kits (Supplementary Table S1). AFP was used as standard control.

### 2.6. Statistical Analysis

All ELISA data are presented as mean ± SD or SEM. The statistical significance of the difference between experimental groups was assessed by unpaired Student *t* test using GraphPad 7.0 software (GraphPad Software Inc., San Diego, CA, USA). Statistical significance was defined as *p* < 0.05. Survival curves were analyzed using the Kaplan–Meier product limit method, and significant differences between each patient group were determined using the Log-rank test. Receiver operating characteristic (ROC) curves were analyzed to calculate sensitivity, specificity and respective areas under the curve (AUC) with 95% confidence intervals (CI) of each candidate marker.

## 3. Results

### 3.1. Gene Selection of Secretory Proteins from Characteristic Molecular Signature of HCC

**Figure 2:**
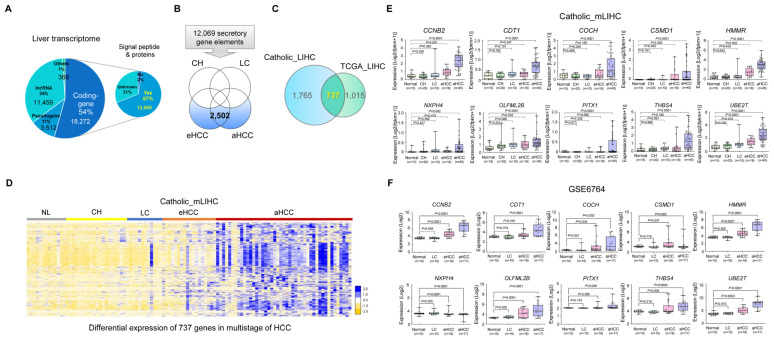
Liver transcriptome scans of characteristic molecular signature in healthy controls and patients with liver disease. (**A**) Pie chart analysis according to gene types and signal peptides of protein coding genes. (**B**) Venn diagram analysis showing genes overexpressed in the early-stage HCC and advanced HCC groups, but not overexpressed in the chronic hepatitis and liver cirrhosis groups. (**C**) Venn diagram analysis of selected genes in (B) from two different RNA-Seq datasets (Catholic_LIHC and TCGA_LIHC). (**D**) Heatmap analysis of 737 HCC-associated gene signatures in Catholic_mLIHC. (**E**,**F**) Expression changes of 10 candidate marker genes in multistage liver disease patients of (**E**) Catholic_mLIHC (cohort 1, *n* = 108) and (**F**) GSE6764 (*n* = 55). Statistically significant differences were determined using Welch’s *t* test.

Comprehensive characterization of the molecular alterations in multistage hepatocellular carcinogenesis is a top priority for identifying biomarkers, particularly for early diagnosis. Thus, to address this need, 108 human hepatic tissues comprising fresh normal liver (NL *n* = 15), CH (*n* = 20), LC (*n* = 10), early-stage HCC (eHCC; high-grade DN *n* = 7 and HCC with Edmonson Grade 1 (G1) *n* = 11) and advanced HCCs (aHCC; G2 *n* = 25 and G3 *n* = 20) were analyzed via whole transcriptome NGS RNA-seq (Catholic_mLIHC, GSE114564). To identify highly expressed secretory molecules within the HCC-specific molecular signature of Catholic_mLIHC, we performed a combined analysis pursuing HCC associated-signaling peptides or proteins (Figure 1 and Supplementary Figure S1). Firstly, liver transcriptome was categorized into coding- and non-coding gene elements, and 18,272 coding gene elements were then subjected to the program SignalP 4.1 (http://www.cbs.dtu.dk/services/SignalP-4.1/ (accessed on 25 April 2016)) to select secretory proteins. These present the potential to define secretory markers that may be detected in the peripheral blood of HCC patients (Figure 2A). From this, 12,069 genes were identified as secretory molecules in liver disease including CH, LC, DNs and multistage HCCs. Next, to identify secretory molecules that are exclusively expressed in HCC, we discarded gene elements that were expressed in CH or LC as indicated in Venn Diagrammatic analysis, resulting in 2502 secretory gene elements remaining (Figure 2B). Further, these 2502 genes were then combined with 1752 HCC-specific gene signatures of TCGA_LIHC and resulted in 737 gene elements as HCC-specific secretory molecules (Figure 2C). Notably, heatmap analysis showed that the majority of 737 genes were gradually increased following progression from eHCC to aHCC (Figure 2D). Next, in order to verify candidate genes in patient samples, an ELISA test was required, so among the 737 genes, genes capable of ELISA testing for the corresponding protein were selected. Among these genes, the top 10 genes with high expression in liver cancer were finally selected. The expression of these 10 candidate gene elements also significantly increased from non-malignant chronic liver disease to aHCC (Figure 2E), and we further validated these genes with another publicly available multistep HCC dataset, GSE6764 (Figure 2F). Lastly, aberrant overexpression of these 10 secretory molecules in HCC was then confirmed with data from TCGA_LIHC (NL *n* = 50, aHCC *n* = 299), ICGC_LIRI (International Cancer Genome Consortium_Liver Cancer-RIKEN, NL *n* = 202, aHCC *n* = 187) and GSE77314 comprising 50 matched pairs of HCC (Supplementary Figure S2).

### 3.2. Serologic Assessment of Candidate Secretory Gene Elements in HCC Patients

**Figure 3:**
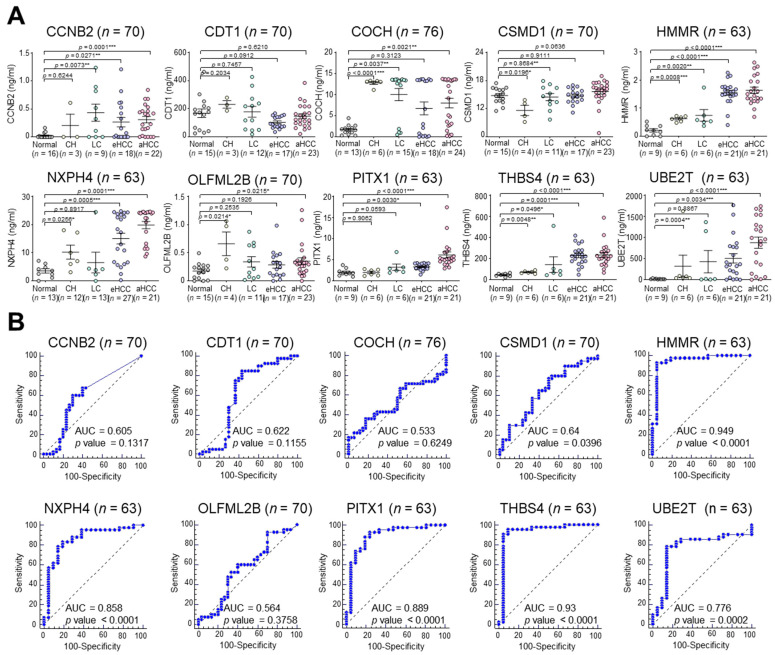
Concentration of 10 secretory proteins in serum in the test set. (**A**) Expression of 10 candidate gene products based on ELISA are presented as an aligned dot plot. Black horizontal lines are means, and error bars are SEs. Mann–Whitney *U* test, *p* < 0.05 *, *p* < 0.01 **, *p* < 0.001 ***. CH, chronic hepatitis B virus infection; LC, liver cirrhosis; eHCC, early-stage hepatocellular carcinoma; aHCC, advanced hepatocellular carcinoma. (**B**) ROC curve analysis of the secretory proteins encoded by the 10 candidate genes. Statistically significant difference of AUC is compared with reference line (AUC = 0.5).

From the computational approaches applied to the Catholic_mLIHC dataset, 10 gene elements were predicted to be potential serologic marker candidates. Therefore, to measure the serum levels of 10 marker proteins in HCC patients, 100 patients comprising 16 normal healthy liver, 13 CH, 15 LC, 35 eHCC and 24 aHCC, were recruited (cohort 2, test set) (Figure 1). Assessments of serum levels of the 10 candidate proteins and AFP were measured using commercially available ELISA (Supplementary Table S1), and all were compared with AFP as the standard HCC diagnostic marker. ELISA analysis showed that the individual protein concentrations of most of the markers were significantly higher in both eHCC and aHCC compared with the corresponding normal with the exception of CDT and CSMD1 (Figure 3A, Supplementary Table S2). Next, comparison analysis of receiver operating characteristic (ROC) curve was performed to determine markers that were better at diagnosing HCC than AFP (Figure 3B, Supplementary Figure S3A,B). Note that we also analyzed the diagnostic performance of des-gamma-carboxy prothrombin (DCP, PIVKA-II) for HCC, but the diagnostic performance of DCP appeared only marginal, and there were no differences in DCP levels between eHCC and CH or LC (Supplementary Figure S3C,D). Thus, to avoid the complexity within our results by adding DCP, we tested the diagnostic ability of the four markers in comparison with AFP only. In the test set of cohort 2, the areas under the curve (AUC) of HMMR (AUC = 0.949, *p* < 0.0001), NXPH4 (AUC = 0.858, *p* < 0.0001), PITX1 (AUC = 0.889, *p* < 0.0001) and THBS4 (AUC = 0.93, *p* < 0.0001) were indicated to be significantly more sensitive and specific than that of AFP (AUC = 0.767, *p* < 0.0001).

**Figure 4:**
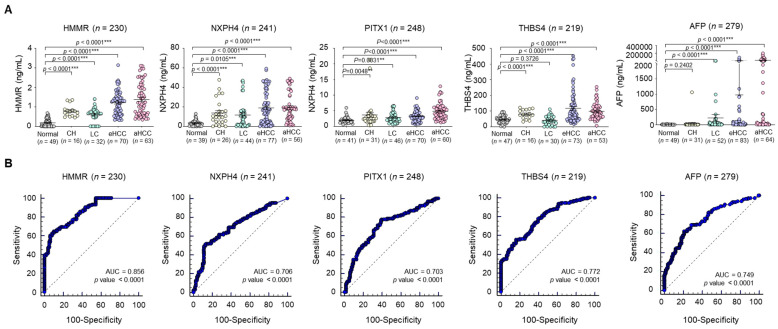
Secretory protein concentration of the four candidate markers in serum in the validation sets. (**A**) Expression of four candidate gene products based on ELISA presented as an aligned dot plot. Black horizontal lines are means, and error bars are SEs. Mann–Whitney *U* test, *p* < 0.01 **, *p* < 0.001 ***. (**B**) The ROC curve analysis of the secretory proteins encoded by the 4 candidate genes in HCC patients versus all control subjects (normal, CH and LC) in the validation cohort. Statistically significant difference of AUC is compared with reference line (AUC = 0.5).

Next, to validate these four as potential serologic markers for HCC detection, large-scale recruitment of HCC patients (a total of 279 patients comprising 49 normal healthy liver, 31 CH, 46 LC, 77 eHCC and 64 aHCC) was undertaken (cohort 3, validation set) for the validation of diagnostic performance (Figure 1). Serum levels of the four markers were significantly higher in both eHCC and aHCC compared with the corresponding normal (Figure 4A, Supplementary Figure S4A). Notably, the HCC group (eHCC and aHCC) also exhibited significantly higher values than the non-tumor groups (normal, CH and LC) for all four markers (Supplementary Figure S4B). We then additionally analyzed the correlation between the levels of AST/ALT and the four markers, but both AST and ALT showed no statistically significant association for each marker (Supplementary Figure S5A,B). In the AUC analysis of cohort 3 (the validation set), HMMR (AUC = 0.856, *p* < 0.0001) and THBS4 (AUC = 0.772, *p* < 0.0001) appeared to be more sensitive and specific than AFP (AUC = 0.749, *p* < 0.0001). ROC curves showed that the optimum diagnostic cutoff for HMMR, NXPH4, PITX and THBS4 was 0.8 ng/mL (sensitivity 80.3%, specificity 91.86%), 7.5 ng/mL (sensitivity 75%, specificity 74.42%), 2.5 ng/mL (sensitivity 80.3%, specificity 66.28%) and 90 ng/mL (sensitivity 57.58%, specificity 90.7%), respectively (Supplementary Table S3). Since in general, the recommended clinical cutoff value for AFP is 20 ng/mL, we chose this as the cutoff value for AFP (sensitivity 52.27%, specificity 84.88%). Likelihood ratios for HMMR, NXPH4, PITX, THBS4 and AFP in the diagnosis of HCC are shown in Supplementary Table S3.

### 3.3. Assessment of HMMR, NXPH4, PITX and THBS4 as a Diagnostic Panel for the Decision of Early HCC

Currently, there is no single serum biomarker with the sensitivity and specificity required for effective HCC screening. Therefore, it is necessary to develop multiprotein serum marker panels to improve specificity in detecting HCC at very early stages of the disease. To investigate the suggested serum markers as panel proteins for the early detection of HCC, we selected patients who underwent screening for all five proteins (HMMR, NXPH4, PITX, THBS4 and AFP) and performed comparative analysis for detecting HCC at the early stage. To this end, 218 patients from the total 379 patients of cohort 2 and cohort 3 were recruited as a comparison set. The clinical characteristics of 132 HCC patients, including 69 with eHCC and 63 with aHCC, are summarized in Supplementary Table S4. Non-tumor groups included 47 NL and 39 patients with non-malignant chronic liver disease (CH *n* = 16, LC *n* = 23).

**Figure 5:**
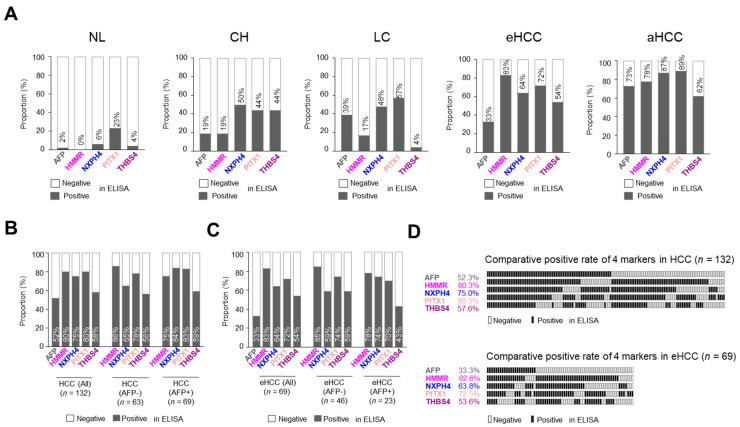
Diagnostic outcomes of four serum markers for the diagnosis of HCC and early-stage HCC. (**A**) The rate of patients with positive AFP and markers in each liver disease status. (**B**) The rate of patients with positive AFP and markers in all patients with HCC and for four markers according to AFP status in all patients with HCC. (**C**) The rate of positive patients for AFP and markers in patients with early-stage HCC and for markers by AFP status in patients with early-stage HCC. ROC, receiver operating characteristics. HCC, hepatocellular carcinoma. NL, normal liver. CH, chronic hepatitis B virus infection. LC, liver cirrhosis. (**D**) The bar chart showing rate with positive AFP and markers in all patients with HCC (**upper** panel) and patients with early-stage HCC (**lower** panel).

In the assessment of differential diagnostic accuracy, serum HMMR had better AUC, sensitivity, specificity, accuracy, positive predictive value (PPV), negative predictive value (NPV) and odds ratio in patients with HCC compared to the non-tumor, non-malignant chronic liver disease and liver cirrhosis groups, respectively (Table 1, Supplementary Figure S6A–C). Notably, we also observed that serum HMMR and THBS4 had better AUC, sensitivity, specificity, accuracy, PPV, NPV and odds ratio in patients with eHCC compared with the non-tumor, non-malignant chronic liver disease and liver cirrhosis groups, indicating these two markers are more appropriate for early diagnosis of HCC than AFP (Table 1, Supplementary Figure S6D–F). Next, we counted the positive rates of these four markers and compared them with APF in multistage liver disease (Figure 5A). The positive rate of AFP was 2% in NL, and four markers exhibited 0~6%, except PITX1 which was 23%. The positive rate of AFP was 73% in aHCC, and the four markers ranged from 62% to ~89%. On the other hand, the positive rate of AFP in eHCC was only 33%, whereas all four markers exhibited rates of 54~83%. Again, although the positive rates of these four markers in non-malignant chronic liver disease (CH and LC) varied, they showed a better performance in the positive rate found for eHCC patients. To support these results, the positive rates of 132 HCC (eHCC–aHCC) patients were re-analyzed. We compared and analyzed the diagnosis rate of liver cancer with four new markers by classifying patients with higher values as positive and negative below the AFP cut-off value (20 ng/mL) in the ELISA test. As expected, the positive rate of the four markers showed a relatively better (58~80%) performance than APF (52%). It is noteworthy that all four demonstrated 56~86% positive values in AFP negative patients (Figure 5B). These four markers also exhibited better results in just eHCC analysis (*n* = 69). AFP was positive in only 33% of eHCC patients, but the panel proteins were positive in 54~83% (Figure 5C). Furthermore, they demonstrated 59~85% detection in the AFP negative eHCC patients. These results were recapitulated by each sample score in the detection of HCC in 132 HCC or 69 eHCC patients (Figure 5D). Detecting HCC or even eHCC using a four-marker combination showed 100% positive detection, whereas AFP alone exhibited 52.3% positive detection in HCCs and 33.3% positive detection in eHCC. These results strongly suggest that serum HMMR, NXPH4, PITX and THBS4 can significantly improve HCC diagnosis and, strikingly, early diagnosis.

### 3.4. Identification of Four Markers and AFP Combinations for Diagnosis of HCC with 100% Accuracy

**Figure 6:**
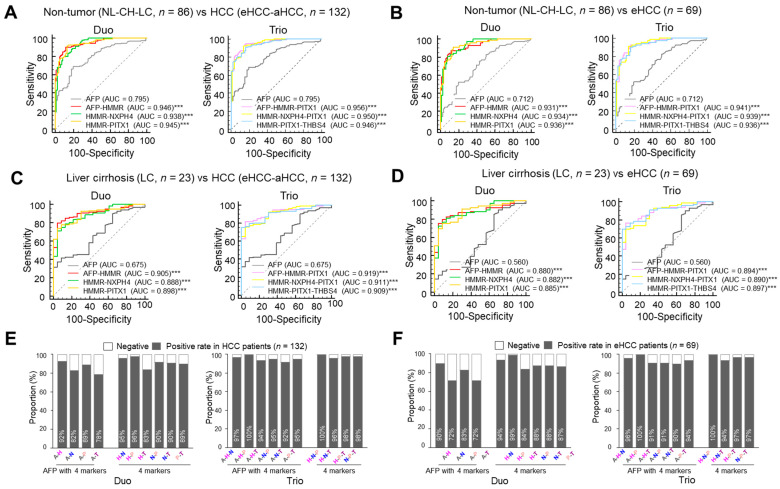
Combinations of AFP with four serum markers for the diagnosis of HCC and early-stage HCC. (**A**) ROC curve of AFP or combination of two markers for patients with all HCC (*n* = 132) versus all controls (*n* = 86). ROC curve of AFP or combination of three markers for patients with all HCC (*n* = 132) versus all controls (*n* = 86). (**B**) ROC curve of AFP or combination of two markers for patients with early-stage HCC (*n* = 69) versus all controls (*n* = 86). ROC curve of AFP or combination of three markers for patients with early-stage HCC (*n* = 69) versus all controls (*n* = 86). (**C**) ROC curve of AFP or combination of two markers for patients with all HCC (*n* = 132) versus liver cirrhosis (*n* = 23). ROC curve of AFP or combination of three markers for patients with all HCC (*n* = 132) versus liver cirrhosis (*n* = 23). (**D**) ROC curve of AFP or combination of two markers for patients with early-stage HCC (*n* = 69) versus liver cirrhosis (*n* = 23). ROC curve of AFP or combination of three markers for patients with early-stage HCC (*n* = 69) versus liver cirrhosis (*n* = 23). (**E**) The rate of positive patients for AFP in pairs with new markers or pairs of new markers in all patients with HCC. The rate of positive patients for triplet combinations of AFP with new markers or triplets of the new markers in all patients with HCC. (**F**) The rate of positive patients for pairs of AFP with new markers or pairs of new markers in patients with early-stage HCC. The rate of positive patients for triplet combinations of AFP with new markers, or new marker triplets in patients with early-stage HCC. *p* < 0.001 ***.

We next investigated whether we could identify combinations of our panel with or without AFP that would demonstrate 100% accuracy in the diagnosis of HCC patients in the comparison set. In the assessment of differential diagnostic accuracy, APF-HMMR, HMMR-NXPH4 and HMMR-PITX1 duos show better AUC, sensitivity, specificity, accuracy and other values in patients with HCC or eHCC compared with non-tumor cases (Table 2). In a comparison of marker triplets, AFP-HMMR-PITX, HMMR-NXPH4-PITX1 and HMMR-PITX1-THBS4, all exhibited better AUC, sensitivity, specificity, accuracy and other values in patients with HCC or eHCC compared with non-tumor cases. All of these combinations were significantly superior to AFP alone (Figure 6A,B). In addition, the ROC comparative analysis of liver cirrhosis also showed superior diagnostic results compared to AFP (Figure 6C,D). Next, we investigated positive rates of pair and triplet marker combinations with or without AFP in the same set. No combinations of AFP with marker pairs reached 100% accuracy for HCC or eHCC patients. However, AFP-HMMR-PITX and HMMR-NXPH4-PITX1 triplets reached 100% accuracy in the diagnosis of both HCC and eHCC patients (Figure 6E,F). Overall, the data suggest that these are the optimal combinations for reaching 100% accuracy in the diagnosis of HCC.

When we designed a selection strategy to identify secretory proteins as novel diagnostic serologic markers, the initial step was to assess the characteristic molecular signature of multistep hepatocellular carcinogenesis and to recapitulate large-scale gene elements that exhibited increased expression during progression from chronic disease to overt cancer. Thus, to investigate the clinical relevance, we investigated alteration frequency of these four marker genes in the TCGA_LIHC dataset. Of the HCC patients, *HMMR* was overexpressed (>2-fold change) in 347 cases of 371 (94%) compared to the mean value of the healthy group (*n* = 50). NXPH4, PITX1 and THBS4 exhibited 89%, 74% and 94%, respectively (Supplementary Figure S7A). Kaplan–Meier survival analysis of the TCGA_LIHC showed that both overall survival (OS) and disease-free survival (DFS) rates of HCC patients with overexpression of all four markers were significantly lower than those of HCC patients with normal expression (Supplementary Figure S7B). Kaplan–Meier survival analysis of the TCGA_LIHC for individual markers also showed that the OS rate of HCC patients with overexpression in each marker gene was significantly lower than that of HCC patients with normal expression, except *THBS4* (Supplementary Figure S7C*)*. These results suggest that *HMMR*, *NXPH4*, *PITX1* and *THBS4* are very selective and potential makers for the diagnosis of liver cancer patients.

## 4. Discussion

Identification of novel serum biomarkers is an important goal for cancer diagnosis, and it is particularly important for diagnosis and examination in early cancer [15]. One of the critical limitations in the development of new strategies for cancer diagnosis and prevention is the deficiency of insight regarding the essential molecular and cellular shift that brings about cancer initiation and progression [16]. Precancerous or premalignant lesions of cancer can help to provide details about the dynamic pathogenesis that precedes development of clinical disease. Global characterization of molecular aberrations in premalignant regions and corresponding alterations in the microenvironment related to development could accelerate the discovery of diagnostic markers for early diagnosis and risk stratification, in addition to contributing to the recognition of preventive interference to reverse or block the development of cancer. Despite the expanded number of studies regarding tracking advanced-stage tumors, studies concerning profiling of genetic alteration that focus on chronic liver disease and pre-cancerous tissues are scarce. The largest barrier impeding the understanding of cancer occurrence and progression and development of early diagnostic devices is the shortage of systematized collection, annotation and profiling of pre-cancerous sites. To address these limitations, we collected 108 human hepatic tissues comprising a spectrum of liver disease including fresh normal liver, CL, LC, DN and different pathological grades of HCCs (G1~3 HCCs) and performed gene expression profiling analysis to discover the characteristic molecular signatures of liver disease (Figure 1).

On the other hand, protein markers detectable in serum are the most appropriate for conventional assessment methods and popular studies of clinical routine. Typically, such examinations are non-invasive, show low reliance on experts, have a low price rate and show high reproducibility, and specimens do not need pretreatments such as reverse transcription, purification or isolation [17,18,19]. Many secretory proteins have been suggested for cancer diagnosis. However, few proteins have been introduced to the clinic in the last few years. This is primarily due to failure to follow strict standards; proteins specifically overexpressed only in cancer and not in adjacent non-tumor tissues; secretory proteins that can be well detected in serum; and rare expression in normal tissues, excluding embryonic tissues [15,20]. Thus, to meet these conditions, we initially established a large-scale molecular signature that is highly specific to HCC development and progression using a multistage HCC transcriptome (Catholic_mLIHC), and from this, secretory peptides or proteins were separated through SignalP 4.1. The 737 gene elements obtained in this way were then subjected to more stringent selection criteria that emphasized marked overexpression in early and advanced HCC and also have not been reported as serum biomarkers for HCC diagnosis. Ten candidate gene elements were suggested through a series of analytic processes, and aberrant expressions of these were then validated with publicly available HCC datasets (TCGA_LIHC, ICGH_LIRI, GSE6764 and GSE77314) (Figure 3, Supplementary Figure S2).

Thus far, AFP is the best HCC marker that has been studied through to phase 5 of biomarker development [21], and in spite of its limited performance, AFP remains the most generally used biomarker. Novel biomarkers for HCC diagnosis have been discovered utilizing advanced genomic, proteomic and metabolomic technologies, and a number of new HCC biomarkers have been identified in the last few decades but have not been widely used in clinical practice yet. Because of the heterogeneous character of cancers, the detective and predictive abilities of biomarkers are limited. Thus, there is no perfect single biomarker for cancer, especially HCC. Therefore, it is absolutely essential to develop combinations of biomarker panels or combinations of biomarkers and clinical parameters to improve the performance of HCC diagnosis. For this reason, we focused on efforts to combine biomarkers to reach a maximum diagnostic and predictive ability. Comparative analysis of the diagnostic values for combinations of four markers with AFP suggested APF-HMMR, HMMR-NXPH4 and HMMR-PITX1 pairs and AFP-HMMR-PITX, HMMR-NXPH4-PITX1 and HMMR-PITX1-THBS4 triplets to be superior in the diagnosis of HCC compared with AFP alone (Table 2). Notably, the AFP-HMMR-PITX and HMMR-NXPH4-PITX1 triplets exhibited 100% accuracy in detecting HCC patients in our comparison set (Figure 6E,F).

Hyaluronan-mediated motility receptor (HMMR), also known as RHAMM (Receptor for Hyaluronan Mediated Motility), was recently reported to promote liver metastasis in an animal model of multistage tumorigenesis [22]. It was also reported that upregulation of HMMR in HCC predicts poor survival. Our results confirmed poor prognosis in HCC patients with HMMR overexpression (Supplementary Figure S7C). Although HMMR has been demonstrated to be upregulated in other cancers, no study has suggested its use as a serologic marker for cancer, especially HCC.

Neurexophilins are secretory neuropeptide-like glycoproteins, and neurexophilin1 and neurexophilin3 are ligands for the presynaptic cell adhesion molecule α-neurexin. Neurexophilin 4, NXPH4, is a secreted glycoprotein, but its function has not been fully described. One recent study has suggested that NXPH4 has a critical role in regulating synapse functions in specific circuits, possibly through interacting with α-neurexin and GABA_A_ receptors [23]. However, no other functional studies have been reported in cancer or for diagnostic usefulness in cancer.

Paired-like homeodomain 1 (PITX1) was originally described as a bicoid-related homeobox transcription factor recruited to regulate the transcription of the pro-opiomelanocortin gene in the adult pituitary and is involved in the differentiation of pituitary cells and in pituitary formation [24]. *PITX1* was later identified as an *hTERT* suppressor gene, located on human chromosome 5 [25]. PITX1 has also been found to be needed to inhibit RAS-induced tumorigenesis; in addition, several studies have shown that PITX1 expression is decreased in colorectal, prostate and lung cancer [26]. Therefore, PITX1 has been implicated as a tumor suppressor in various cancers. However, contrary to previous observations in other cancers, our analysis here showed aberrant overexpression in liver cancer and its association with poor prognosis of HCC patients (Supplementary Figure S7C). Thus, it can be used as a marker for HCC diagnosis.

Thrombospondin 4, THBS4, is an evolutionarily conserved extracellular calcium-binding glycoprotein that is secreted as a pentameric globular complex, becoming part of the extracellular matrix, and is involved in key cellular processes, such as proliferation, attachment, adhesion and migration [27]. Moreover, an increasing number of studies have suggested that THBS4 is associated with the pathophysiology of different types of malignancies. For instance, the tumor-suppressing role of THBS4 on the proliferation of colorectal cancer was reported, but a pro-tumorigenic role for THBS4 was also reported in prostate and gastric caners [28,29]. Recently, overexpression of THBS4 in HCC was reported, but no study on its use as a serologic marker for HCC has been conducted. Collectively, the data presented here demonstrate that these four proteins, HMMR, NXPH4, PITX and THBS4, are novel secretory biomarkers for diagnosing HCC patients at an early stage of HCC development.

HCC is one of the few cancers that are on the rise [30]. Although recent developments in examination and novel drugs have led to advances in the prevention, diagnosis and treatment of liver cancer, clinicians still face challenges in detecting cancer at an early stage. Among HCC patients, the number of early diagnosed patients is only as high as 44%. When HCC patients are diagnosed at later stages, fewer than 16% survive for 5 years, but contrastingly, when HCC patients are diagnosed at an early stage, nearly 70% survive beyond 5 years [31].

## 5. Conclusions

Diagnosing HCC as early as possible is important to improve patient prognosis. A biomarker panel consisting of HMMR, NXPH4, PITX1 and THBS4 was defined and validated as an effective serologic diagnostic tool for the detection of HCC patients among liver disease patients through the use of simple ELISAs. The biomarker panel could identify AFP false-negatives and discriminate patients with early-stage HCC. The diagnostic performance overall was vastly superior to that of AFP alone. We therefore believe this four-marker panel has the potential to be widely used in clinical practice for HCC diagnosis, but further research is required to prove the clinical utility of these promising biomarkers.

## Figures and Tables

**Table 1:** Results for measurement of HMMR, NXPH4, PITX1 and THBS4 serum markers and AFP in the diagnosis of HCC.

	AUC (95% CI)	Sensitivity (%)	Specificity (%)	Accuracy (%)	PPV (%)	NPV (%)	Odds Ratio
HCC (eHCC-aHCC) vs. Non-tumor (NL-CH-LC)							
AFP	0.793	51.13	86.05	64.84	85.00	53.24	6.45
**“HMMR”**	0.914	79.70	91.86	84.47	93.81	74.53	44.31
NXPH4	0.789	74.44	74.42	74.43	81.82	65.31	8.47
PITX1	0.777	79.70	63.95	73.52	77.37	67.07	6.97
THBS4	0.791	57.14	88.37	69.41	88.37	57.14	10.13
HCC (eHCC-aHCC) vs. Non-malignant liver disease (CH-LC)							
AFP	0.717	51.13	71.79	55.81	86.08	30.11	2.66
**“HMMR”**	0.832	79.70	82.05	80.23	93.81	54.24	17.95
NXPH4	0.694	74.44	51.28	69.19	83.90	37.04	3.07
PITX1	0.718	79.70	48.72	72.67	84.13	41.30	3.73
**“THBS4”**	0.735	57.14	79.49	62.21	90.48	35.23	5.17
eHCC vs. Non-tumor (NL-CH-LC)							
AFP	0.71	31.43	86.05	61.54	64.71	60.66	2.83
**“HMMR”**	0.915	81.43	91.86	87.18	89.06	85.87	49.48
NXPH4	0.742	62.86	74.42	69.23	66.67	71.11	4.92
PITX1	0.681	71.43	63.95	67.31	61.73	73.33	4.44
**“THBS4”**	0.748	52.86	88.37	72.44	78.72	69.72	8.52
eHCC vs. Non-malignant liver disease (CH-LC)							
AFP	0.613	31.43	71.79	45.87	66.67	36.84	1.17
**“HMMR”**	0.835	81.43	82.05	81.65	89.06	71.11	20.04
NXPH4	0.648	62.86	51.28	58.72	69.84	43.48	1.78
PITX1	0.606	71.43	48.72	63.30	71.43	48.72	2.38
**“THBS4”**	0.693	52.86	79.49	62.39	82.22	48.44	4.34

AUC: area under the curve. PPV: positive predictive values. NPV: negative predictive values. **“Protein”**: marker superior to AFP.

**Table 2:** Results for measurement of pair or triplet combinations of HMMR, NXPH4, PITX1 and THBS4 serum markers with AFP in the diagnosis of HCC.

HCC (eHCC-aHCC) vs. Non-Tumor (NL-CH-LC)											
	AUC	95% CI	Sensitivity(%)	Specificity(%)	+LR	−LR	Accuracy(%)	PPV(%)	NPV(%)	Odds Ratio	Relative Risk
AFP	0.795	0.735–0.846	52.27	84.88	3.46	0.56	65.14	52.27	84.88	6.15	0.71
AFP-HMMR	0.946	0.907–0.972	90.15	88.37	7.75	0.11	89.45	92.25	85.39	69.57	6.32
HMMR-NXPH4	0.938	0.897–0.966	78.79	91.86	9.68	0.23	83.94	93.69	73.83	41.92	3.58
HMMR-PITX1	0.945	0.906–0.971	92.42	86.05	6.62	0.09	89.91	92.42	86.05	75.23	7.95
AFP-HMMR-PITX1	0.956	0.920–0.979	93.94	84.88	6.21	0.07	90.37	93.94	84.88	87.04	10.10
HMMR-NXPH4-PITX1	0.950	0.912–0.975	92.42	86.05	6.62	0.09	89.91	92.42	86.05	75.23	7.95
HMMR-PITX1-THBS4	0.946	0.907–0.972	90.91	83.72	5.58	0.11	88.07	90.91	83.72	51.43	6.52
**eHCC vs. Non-Tumor (NL-CH-LC)**											
	**AUC**	**95% CI**	**Sensitivity(%)**	**Specificity(%)**	**+LR**	**−LR**	**Accuracy(%)**	**PPV(%)**	**NPV(%)**	**Odds Ratio**	**Relative Risk**
AFP	0.712	0.634–0.782	33.33	84.88	2.21	0.79	61.94	33.33	84.88	2.81	0.62
AFP-HMMR	0.931	0.879–0.965	86.96	88.37	7.48	0.15	87.74	86.96	88.37	50.67	8.31
HMMR-NXPH4	0.934	0.882–0.967	81.16	91.86	9.97	0.21	87.10	81.16	91.86	48.62	5.37
HMMR-PITX1	0.936	0.886–0.969	91.30	86.05	6.54	0.10	88.39	91.30	86.05	64.75	13.09
AFP-HMMR-PITX1	0.941	0.892–0.973	92.75	83.72	5.70	0.09	87.74	92.75	83.72	65.83	15.95
HMMR-NXPH4-PITX1	0.939	0.889–0.971	91.30	86.05	6.54	0.10	88.39	91.30	86.05	64.75	13.09
HMMR-PITX1-THBS4	0.936	0.885–0.969	89.86	83.72	5.52	0.12	86.45	89.86	83.72	45.55	11.04

## Data Availability

The data presented in this study are available on request from the corresponding author.

## Supplementary Materials

The following supporting information can be downloaded at: https://www.mdpi.com/article/10.3390/jcm11082128/s1, Figure S1: Pipeline for identifying potential secretory markers for diagnosing hepatocellular carcinoma; Figure S2: Differential gene expression of 10 secretory molecules; Figure S3: Concentration of AFP and PIVKA-II in the test sets; Figure S4: The concentration of 4 secretory proteins in serum in the validation set; Figure S5: Comparative analysis of four new markers with AST/ALT in liver cancer patients; Figure S6: Diagnostic efficiency of AFP, HMMR, NXPH4, PITX1, and THBS4 for HCC; Figure S7: mRNA expression and prognostic power of HMMR, NXPH4, PITX1 and THBS4 in TCGA_LIHC; Table S1: List of ELISA kit for 10 candidate markers testing; Table S2: The mean concentration of 10 markers in serum; Table S3: The optimum diagnostic cutoff values of 4 serum markers; Table S4: The clinical characteristics of HCC patients in the comparison set.
