# Supplementary material for: Serum Proteins, HMMR, NXPH4, PITX1 and THBS4; A Panel of Biomarkers for Early Diagnosis of Hepatocellular Carcinoma
Source: J Clin Med. 2022 Apr 11;11(8):2128. doi: 10.3390/jcm11082128 (PMC9027255; doi:10.3390/jcm11082128)
Supplement: Supplementary file 1 [file jcm-11-02128-s001.zip › jcm-1644777-supplementary.pdf]

Supporting Information

Supplementary Figures

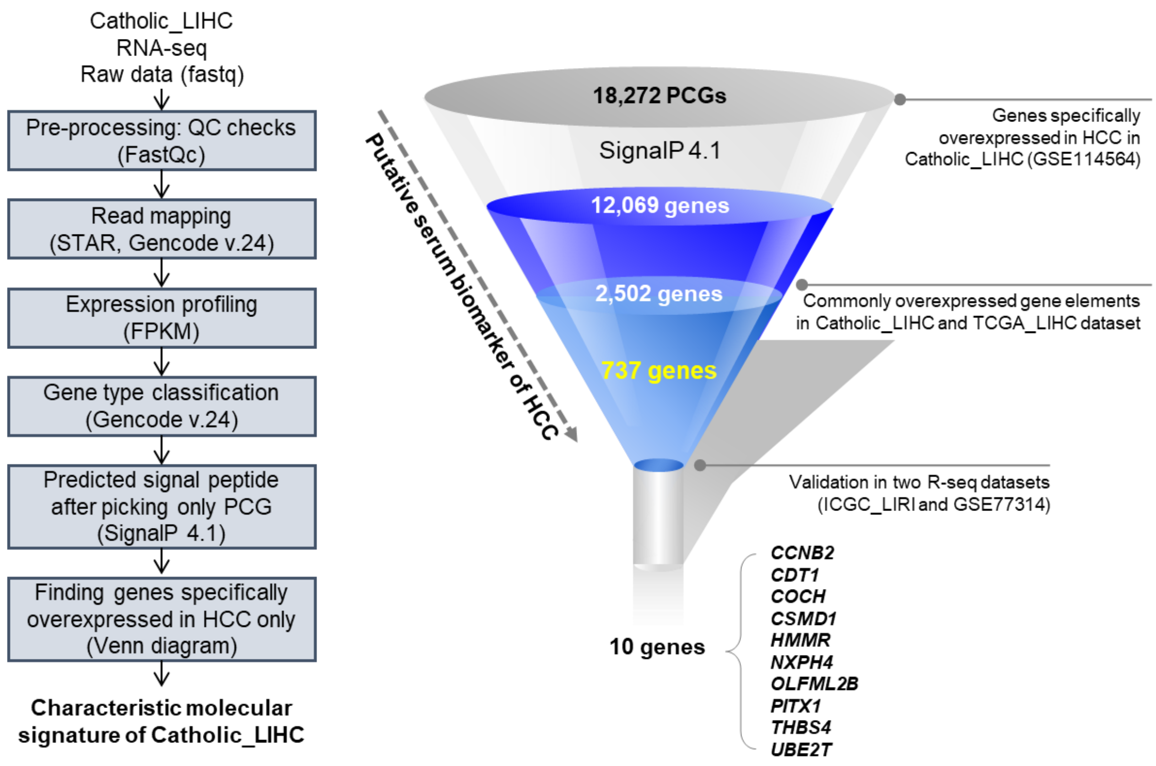

*PCG: Protein coding gene
*CCNB2: Cyclin B2
*CDT1: Chromatin Licensing And DNA Replication Factor 1
*COCH: Cochlin
*CSMD1: CUB And Sushi Multiple Domains 1
*HMMR: Hyaluronan Mediated Motility Receptor
*NXPH4: Neurexophilin 4
*OLFML2B: Olfactomedin Like 2B
*PITX1: Paired Like Homeodomain 1
*THBS1: Thrombospondin 4
*UBE2T: Ubiquitin Conjugating Enzyme E2 T

Supplementary Figure S1. Pipeline for identifying potential secretory markers for diagnosing hepatocellular carcinoma.

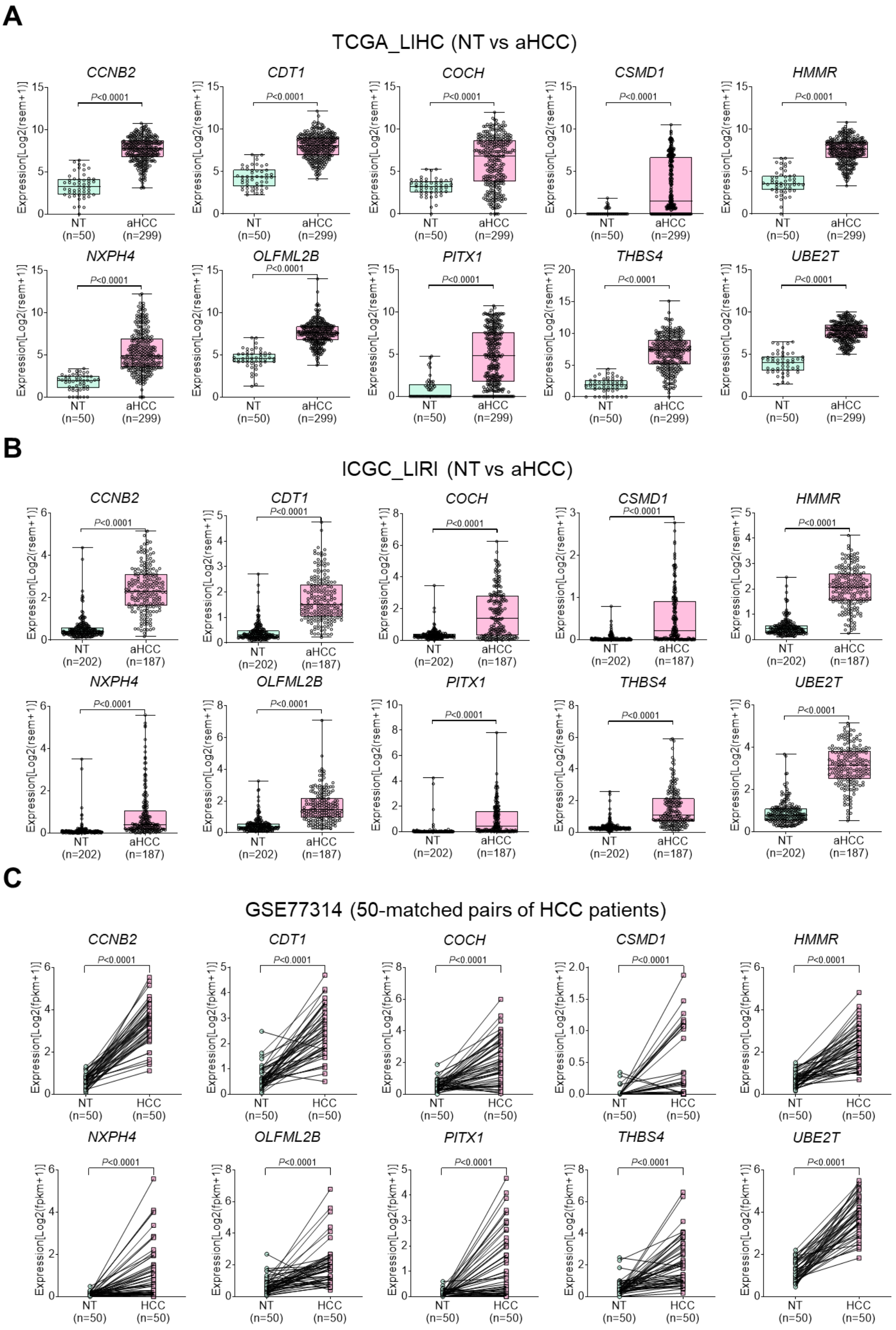

Supplementary Figure S2. Differential gene expression of 10 secretory molecules (**A** and **B**) Differential gene expression of 10 secretory marker genes in HCC patients with non-tumor

and advanced HCC from (**A**) TCGA_LIHC and (**B**) ICGC_LIRI dataset. Unpaired student's t test, $P < 0.0001$*** (**C**) Differential gene expression of 10 secretory candidate markers in 50-matched pairs of HCCs from GSE77314. Paired student's t test, $P < 0.001$***.

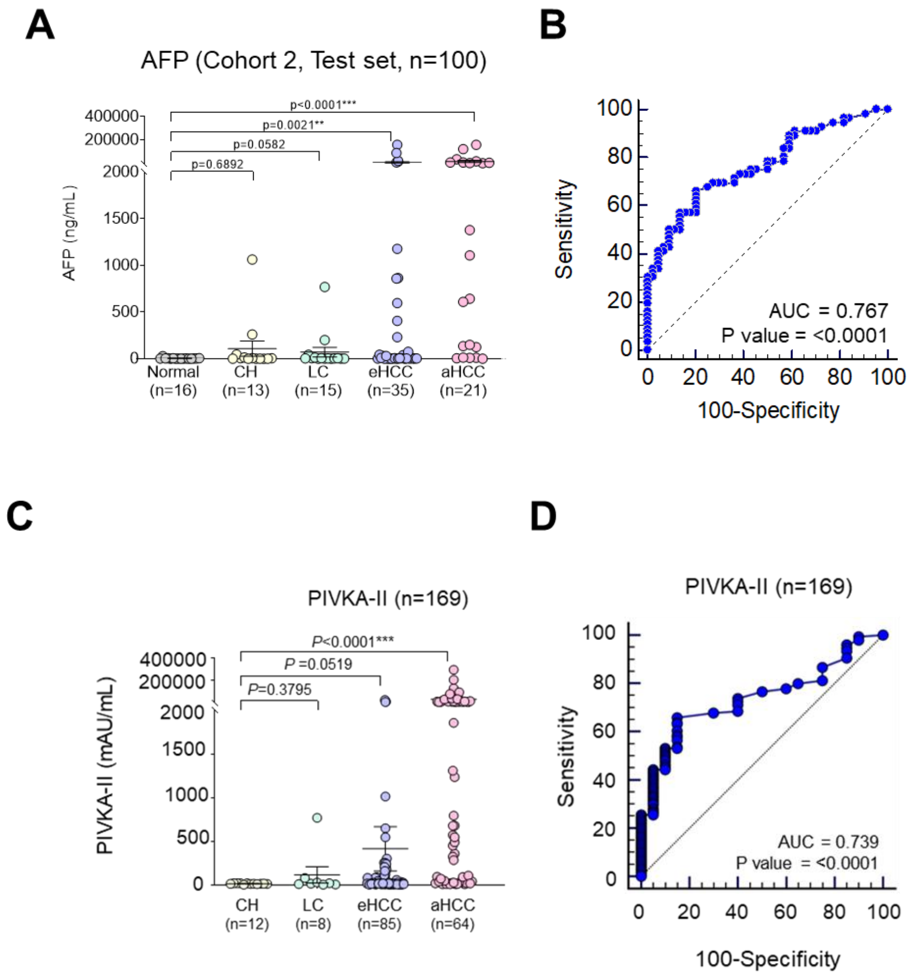

Supplementary Figure S3. Concentration of AFP and PIVKA-II in the test sets. (**A** and **C**) AFP (**A**) and PIVKA-II (**C**) levels in serum was measured by ELISA in the test sets. Mann-Whitney U test, P < 0.01**, P < 0.0001***. (**B** and **D**) The receiver operating characteristic (ROC) curve analysis of AFP (**B**) and PIVKA-II (**D**). Statistically significant difference of AUC is compared with reference line (AUC=0.5).

A

	HMMR		NXPH4		PITX1		THBS4		(ng/ml)
	Non-tumor (n=97)	HCC (n=133)	Non-tumor (n=109)	HCC (n=133)	Non-tumor (n=118)	HCC (n=130)	Non-tumor (n=93)	HCC (n=126)	
Minimum	0.00	0.32	0.00	0.00	0.36	0.62	0.00	6.74	
25% Percentile	0.00	0.64	1.32	4.83	1.48	2.47	29.23	54.07	
Median	0.43	1.24	4.49	16.38	2.15	3.49	46.57	81.91	
75% Percentile	0.67	1.74	12.50	31.84	3.23	5.32	72.63	138.00	
Maximum	1.41	3.14	47.80	59.30	21.37	12.85	122.60	452.60	
Mean	0.42	1.29***	9.18	18.95***	2.74	3.96***	49.53	109.60***	
Std. Deviation	0.38	0.73	11.71	16.06	2.67	2.32	29.86	84.67	
Std. Error of Mean	0.04	0.06	1.12	1.39	0.25	0.20	3.10	7.54	
Lower 95% CI of mean	0.34	1.17	6.95	16.20	2.25	3.56	43.38	94.66	
Upper 95% CI of mean	0.49	1.41	11.40	21.71	3.23	4.36	55.68	124.50	

B

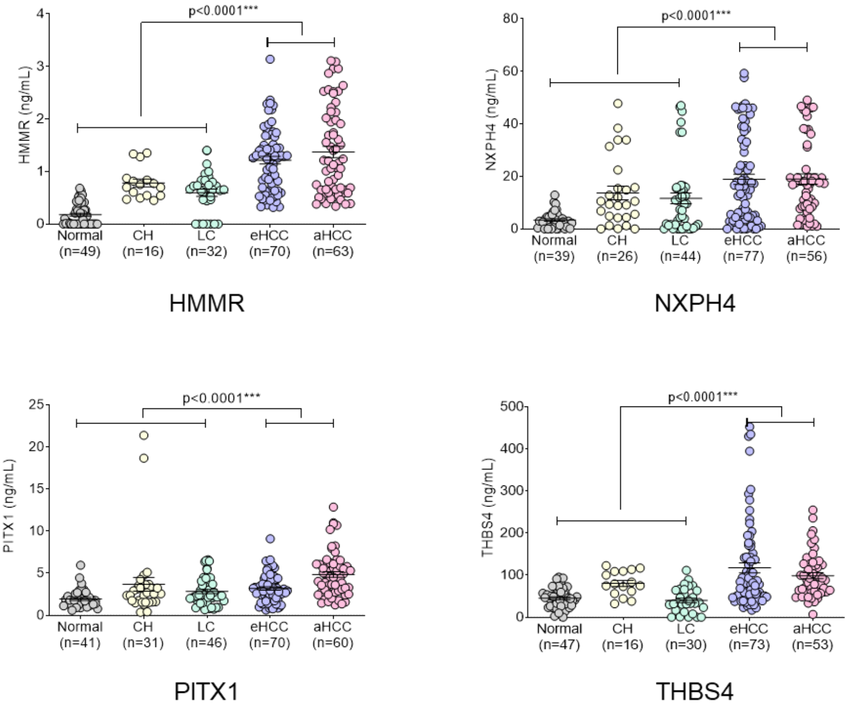

Supplementary Figure S4. The concentration of 4 secretory proteins in serum in the validation set. (**A**) The concentration of 4 markers compared with that of AFP in serum is listed in the table. Mann-Whitney U test, P < 0.0001***. (**B**) Expressions of 4 marker proteins based on ELISA test are presented as aligned dot plot. Mann-Whitney U test, P < 0.001***.

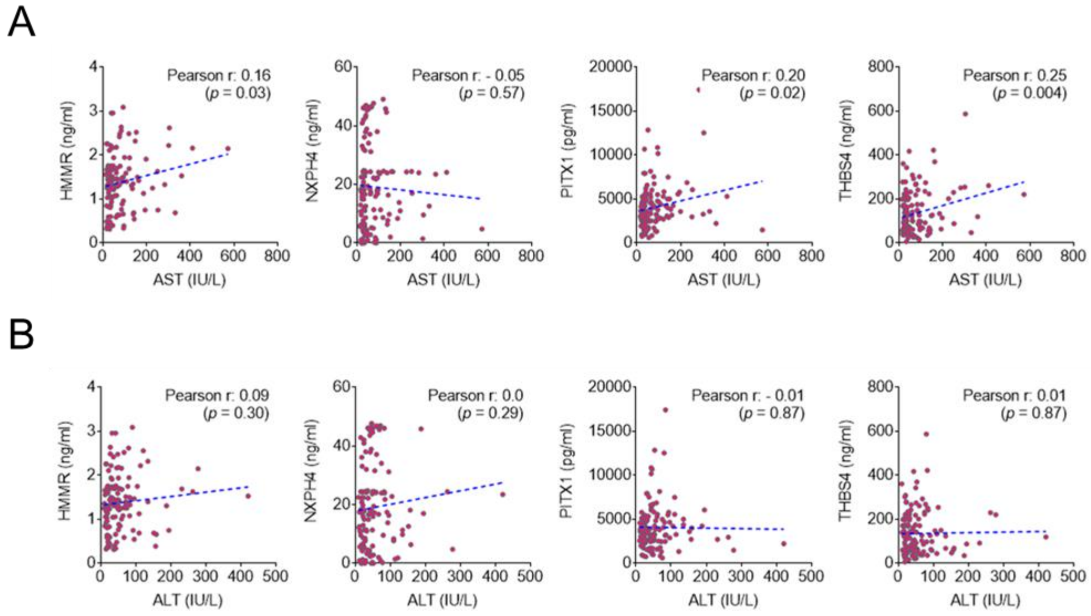

Supplementary Figure S5. Comparative analysis of four new markers with AST/ALT in liver cancer patients. Correlation of AST (**A**) or ALT (**B**) with each of four markers in liver cancer patients. Pearson's coefficient test.

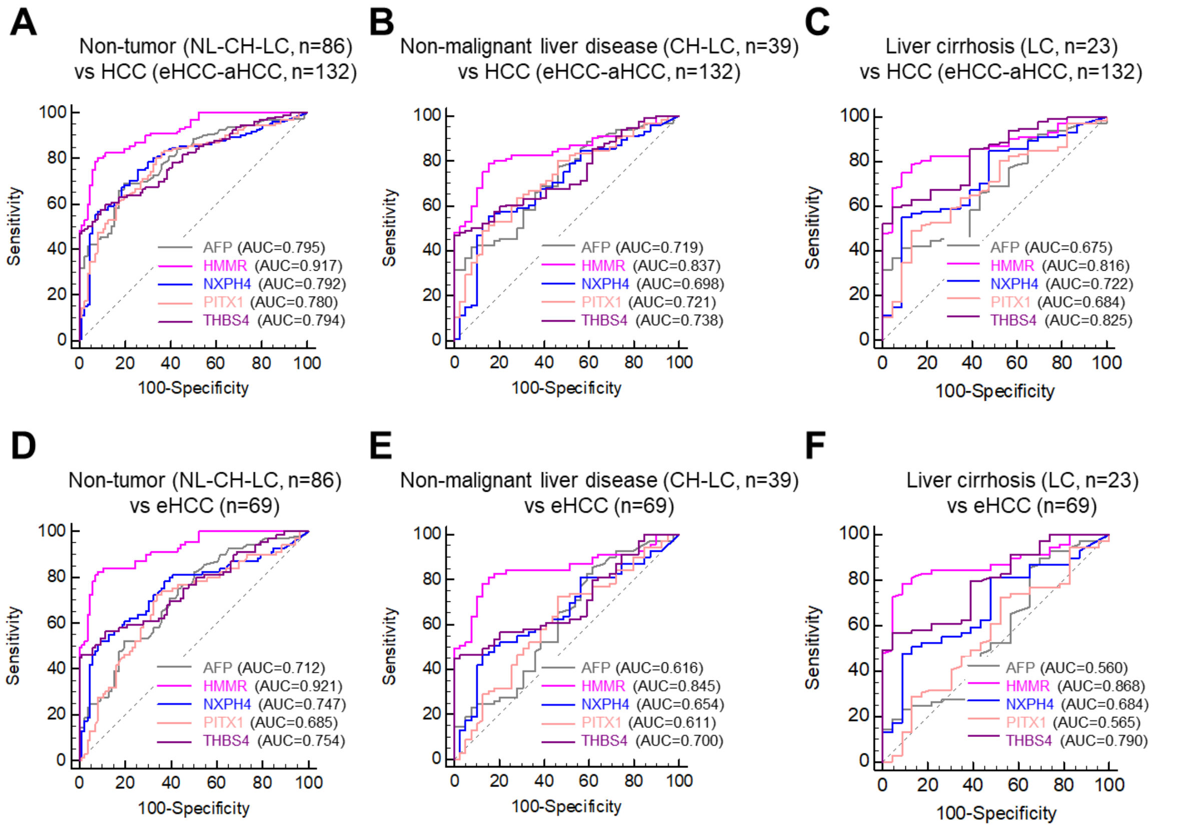

Supplementary Figure S6. Diagnostic efficiency of AFP, HMMR, NXPH4, PITX1, and THBS4 for HCC. (**A**, **B**, **C**) ROC curve for AFP and 4 markers for all patients with HCC versus non-tumor, liver disease or liver cirrhosis group. (**D**, **E**, **F**) ROC curve of AFP and 4 markers for patients with early HCC versus non-tumor, non-malignant liver disease or liver cirrhosis group.

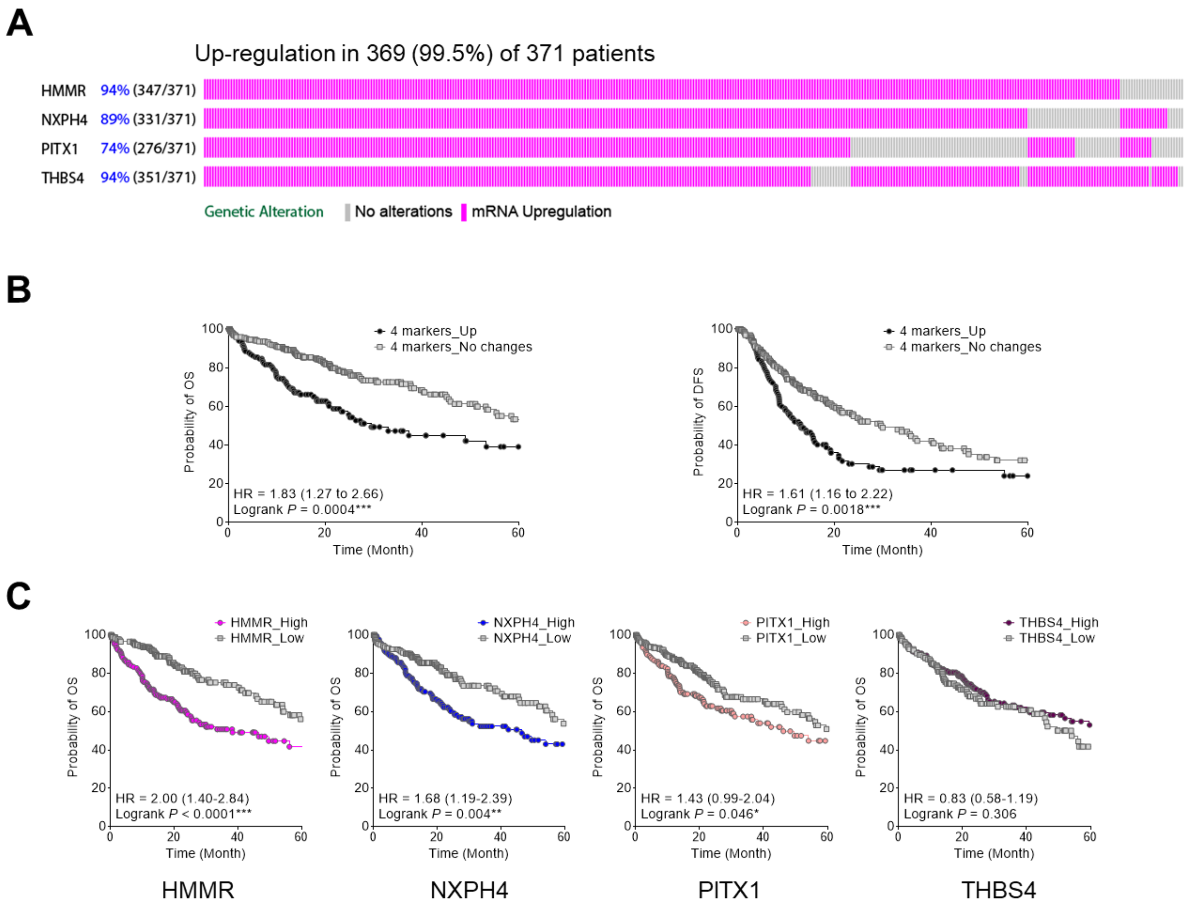

Supplementary Figure S7. mRNA expression and prognostic power of HMMR, NXPH4, PITX1 and THBS4 in TCGA_LIHC. (**A**) The bar chart showing each gene expression of patients with HCC over 2-fold up-regulated compared to non-tumor group in TCGA_LIHC. (**B**) Kaplan-Meier survival curves with mRNA expression of 4 markers (HMMR, NXPH4, PITX1, and THBS4) in HCC patients from TCGA_LIHC for overall (left panel) and disease free (right panel) survivals. (**C**) Kaplan-Meier survival curves with each mRNA expression of HMMR, NXPH4, PITX1, and THBS4 in HCC patient from TCGA_LIHC for overall survival.

Supplementary Tables

Supplementary Table S1. List of ELISA kit for 10 candidate markers testing

Gene	Protein	Company	Cat. No.	Detection range	Dilution factor
AFP	α-fetoprotein	Siemens	110763	1.3 ~ 1000 ng/ml	1 : 100
CCNB2	Cyclin B2	MyBioSource	MBS2019553	0.156 ~ 10 ng/ml	1 : 1
CDT1	DNA replication factor Cdt1	CUSABIO	CSB-EL005125HU	0.0235 ~ 1.5 ng/ml	1 : 1
COCH	Cochlin	MyBioSource	MBS9300263	0.25 ~ 8 ng/ml	1 : 1
CSMD1	CUB and Sushi multiple domains 1	MyBioSource	MBS7206464	0.5 ~ 10 ng/ml	1 : 2
HMMR	Hyaluronan mediated motility receptor	CUSABIO	CSB-EL010582HU	0.156 ~ 10 ng/ml	1 : 1
NXPH4	Neurexophilin 4	MyBioSource	MBS9311035	0.625 ~ 20 ng/ml	1 : 1
OLFML2B	Olfactomedin-like protein 2B	CUSABIO	CSB-EL016326HU	0.0625 ~ 4 ng/ml	1 : 1
PITX1	Paired like homeodomain 1	CUSABIO	CSB-EL018042HU	0.0125 ~ 0.8 ng/ml	1 : 10
THBS4	Thrombospondin 4	CUSABIO	CSB-EL023490HU	7.8 ~ 500 ng/ml	1 : 1
UBE2T	Ubiquitin conjugating enzyme E2 T	MyBioSource	MBS9338696	0.625 ~ 20 ng/ml	1 : 100

Supplementary Table S2. The mean concentration of 10 markers in serum

	The mean concentration of 10 markers				
	NL	CH	LC	eHCC	aHCC
AFP (ng/ml)	4.84±6.59	108.5±295.70	71.51±199.70	8604±30581	17566±42440
CCNB2 (ng/ml)	0.02±0.05	0.20±0.35	0.43±0.46	0.27±0.36	0.31±0.26
CDT1 (ng/ml)	0.17±0.12	0.23±0.07	0.18±0.13	0.10±0.04	0.15±0.08
COCH (ng/ml)	1.72±1.14	12.78±0.82	10.03±5.59	6.74±6.39	8.03±5.61
CSMD1 (ng/ml)	14.80±1.70	11.65±2.19	14.48±2.52	14.72±1.57	15.66±2.53
OLFML2B (ng/ml)	0.21±0.18	0.62±0.10	0.74±0.50	1.54±0.42	1.64±0.52
HMMR (ng/ml)	3.54±2.31	10.23±6.13	6.52±8.94	15.02±8.68	19.83±5.96
NXPH4 (ng/ml)	0.18±0.14	0.66±0.42	0.34±0.31	0.28±0.24	0.35±0.30
PITX1 (ng/ml)	2.04±0.87	1.99±0.73	3.24±1.82	3.31±0.73	6.14±3.30
THBS4 (ng/ml)	45.36±13.50	70.96±12.58	141.80±181.70	229.40±93.05	233.60±113.20
UBE2T (ng/ml)	16.14±9.14	319.90±639.40	426.10±646.60	505.50±501.00	877.20±603.50

Supplementary Table S3. The optimum diagnostic cutoff values of 4 serum markers

	Criterion	Sensitivity (%)	95% CI	Specificity (%)	95% CI	+LR	-LR
HMMR	0.8 ng/ml	80.30	72.5 - 86.7	91.86	83.9 - 96.7	9.87	0.21
NXPH4	7.5 ng/ml	75.00	66.7 - 82.1	74.42	63.9 - 83.2	2.93	0.34
PITX1	2.5 ng/ml	80.30	72.5 - 86.7	66.28	55.3 - 76.1	2.38	0.30
THBS4	90 ng/ml	57.58	48.7 - 66.1	90.70	82.5 - 95.9	6.19	0.47

CI: Confidence interval

LR: Likelihood-ratio

Supplementary Table S4. The clinical characteristics of HCC patients in the comparison set

Variable		HCC patients of the Comparison set (n=132)	
		No.	Percentage (%)
Sex	Female	28	21.2
	Male	104	78.8
Age (years)	≤65	72	54.5
	>65	60	45.5
Cause	HBV	81	61.4
	HCV	16	12.1
	Alcohol	17	12.9
	Others	18	13.6
AST (U/L)	≤80	100	75.8
	>80	32	24.2
ALT (U/L)	≤60	96	72.7
	>60	36	27.3
T-Bil (mg/dL)	≤1.2	101	76.5
	>1.2	31	23.5
Albumin (g/dL)	≤3.5	57	43.2
	>3.5	75	56.8
PT (INR)	≤1.2	89	68.0
	>1.2	43	31.8
Child-Pugh classification	A	107	81.1
	B	20	15.2
	C	5	3.8
AFP (ng/mL)	≤20	64	48.5
	>20	68	51.5
Tumor size (cm)	<2	23	17.4
	≥2	66	50
	Unknown	43	32.6
Tumor number	1	53	40.2
	2-3	25	18.9
	4 or more	13	9.8
	Unknown	41	31.1
Tumor Staging (mUICC)	Ⅰ	27	20.5
	Ⅱ	43	32.6
	Ⅲ	27	20.5
	Ⅳ	15	11.4
	Ⅴ	20	15.2

AST, Aspartate transaminase

ALT, Alanine transferase

T-Bil, T-bilirubin

PT (INR), Prothrombin time and international normalized ratio
